# Heart rate reduction as a marker to optimize carvedilol treatment and enhance myocardial recovery in pediatric dilated cardiomyopathy

**DOI:** 10.3389/fphys.2022.1001752

**Published:** 2022-12-02

**Authors:** Rachele Adorisio, Giuseppe Pontrelli, Nicoletta Cantarutti, Elisa Bellettini, Martina Caiazza, Erica Mencarelli, Giuseppe Limongelli, Daniela Poli, Fabrizio Drago, Richard Kirk, Antonio Amodeo

**Affiliations:** ^1^ Advanced Cardiovascular Therapies Unit- Heart Failure, Transplant and Mechanical Assist Device, Department of Cardiology, Cardiac Surgery and Heart Lung Transplant, Bambino Gesù Pediatric Hospital and Research Institute, Rome, Italy; ^2^ Quality Team of Clinical Study- Director, Department of Pediatrics, Bambino Gesù Pediatric Hospital and Research Institute, Rome, Italy; ^3^ Cardiology and Cardiac Arrhythmias Unit, Department of Cardiology, Cardiac Surgery and Heart Lung Transplant, Bambino Gesù Pediatric Hospital and Research Institute Bambino Gesù Pediatric Hospital and Research Institute, Rome, Italy; ^4^ Genetic Disease and Rare Cardiovascular Disorder, Luigi Vanvitelli University, AORN Colli, Monaldi Hospital, Naples, Italy; ^5^ Heart Failure, Transplant and Mechanical Assist Device, Department of Cardiology, Cardiac Surgery and Heart Lung Transplant, Bambino Gesù Pediatric Hospital and Research Institute Bambino Gesù Pediatric Hospital and Research Institute, Rome, Italy; ^6^ Centro Cardiologico Pediatrico del Mediterraneo—Bambino Gesù Hospital—Taormina—Italia, Villagonia, Italy

**Keywords:** heart rate, dilated cardiomyopathy, children, heart failure, carvedilol

## Abstract

**Introduction:** An elevated heart rate is associated with an increased risk of death or cardiac transplant in children with dilated cardiomyopathy (DCM). Whether heart rate is a clinical marker to address therapy, is poorly investigated in children.

**Aim:** To investigate the relationship between heart rate reduction (HRR) and left ventricular ejection fraction (LVEF) in DCM, treated with carvedilol.

**Methods:** This is a multi center retrospective analysis conducted on all children with DCM (aged <18 years) between 2013 and 2020, with LVEF <40% and treated with carvedilol. Carvedilol was up titrated to the maximal tolerated dose or to 1 mg/kg/day. Echocardiographic data on left ventricular function and dimension were collected. The relationship between HRR and LVEF, left ventricular end-diastolic (LVEDd) and end-systolic diameter (LVESd) was assessed before and after HRR with carvedilol, using regression analysis.

**Results:** 100 patients were enrolled (M: 51%; age 7 ± 8 years). The mean LVEF was 30.2 ± 10% before treatment and 43.7 ± 9.6% after treatment, at the maximum therapeutic dose (*p* < 0.0001). There was a positive relationship between HRR and increase in LVEF (*R* ^2^ = 0.06, *p* = 0.014). A HRR of >20% correlated with an improvement in LVEF >13%. At 3 years follow up, HRR demonstrated a significant reduction of LVESd (R2 = 0.1, *p* = 0.003) LVEDd (R2 = 0.07, *p* = 0.008) and LVEF recovery up to 15% (*p* < 0.0001). No deaths or heart transplant occurred during follow-up.

**Conclusion:** This study demonstrates that HRR is safe and improvement in LVEF is related to the degree of HRR. The magnitude of LVEF improvement was enhanced by a major reduction in HR. It provides evidence that HRR could be used as a clinical marker to treat HF in children.

## Introduction

The beneficial effects of beta blockers in chronic heart failure (CHF), especially in adults, are well recognized and this class of drugs is currently recommended to improve prognosis Packer et al., 1998. However the routine use of beta blockers is weak in paediatric CHF. To date, the only clinical trial, which used carvedilol, in children failed to show any beneficial effect (Shaddy, et al., 2007). Despite this lack of evidence in children, carvedilol use is widespread but its management varies among centers. Beta blockers present a marked individual response, making its management and obtaining unequivocal results challenging. It remains unclear in paediatric CHF what represents the optimal dose, what is the target to assess the efficacy and the safety for each individual patient.

Heart rate is one of the main markers of heart failure at any age (Fox, et al., 2007; Rossano, et al., 2020). Recently, Rossano et al. showed that in children with dilated cardiomyopathy (DCM), elevated heart rate (HR) was associated with an increased risk of death and cardiac transplant. Reduction in HR, may therefore be more important than the achievement of target doses, as published in the major clinical trials in adult population (Lechat et al., 1998; Fox et al., 2008; McAlister et al., 2009; Packer et al., 2001; Packer et al., 2002; Swedberg et al., 2010).

The aim of this study is to investigate the relationship between heart rate reduction (HRR) and left ventricular function in pediatric patients in chronic heart failure, with DCM, treated with carvedilol.

## Methods

Data were reviewed retrospectively from hospital database from 2013 to 2019 of all consecutive pediatric patients affected by DCM and left ventricular (LV) dysfunction referred to Bambino Gesù Children Hospital, Rome, Monaldi Hospital, Napoli, and Centro Cardiologico Mediterraneo Taormina. DCM was defined by the presence of LV dilatation (>2 SD) and systolic dysfunction in the absence of abnormal loading conditions (hypertension, valve disease) or coronary artery disease sufficient to cause global systolic impairment (Pinto et al., 2016). Inclusion criteria for the analysis were: 1. age at clinical presentation <18 years; 2. Ejection fraction <40% 3. ACE inhibitors [i.e. captopril, enalapril, ramipril] started since at least 72 h before 4. Allowed concomitant treatment included diuretics and mineral corticoid therapy. 5. BNP <1,000 pg/ml.

**FIGURE 1:**
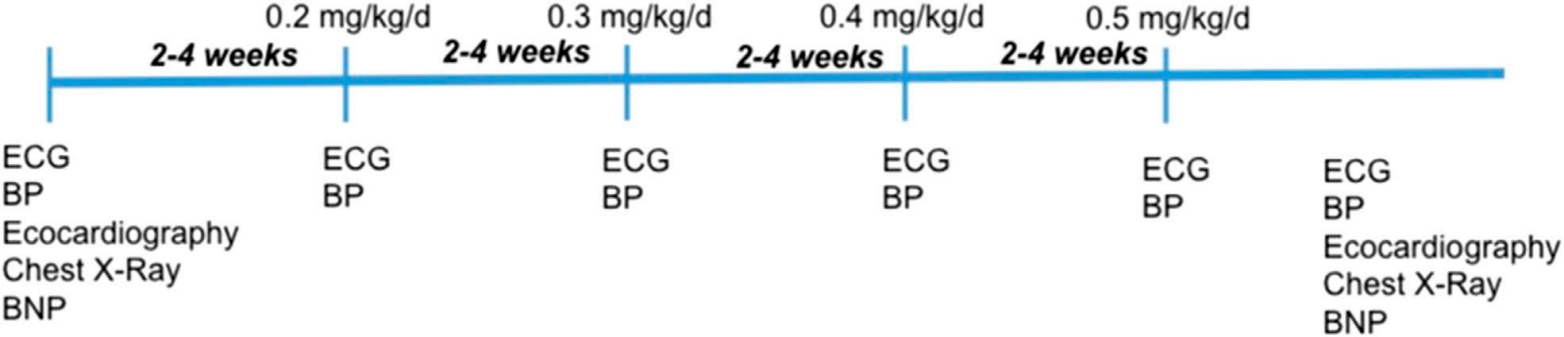
Uptitration of carvedilol during the follow up. Each dose was uptitrated every 2–4 weeks. ECG: electrocardiogram; BP: Blood Pressure.

Carvedilol was up titrated starting from 0.1 mg/kg/dose BID to the maximum tolerated dose every 2–4 weeks and/or maximum dosage of 0.5 mg/kg/dose BID. The maximum tolerated dose was defined according to the onset of symptomatic hypotension and/or bradycardia (<60 bpm) (Figure 1).

Data were collected for each patient at baseline, at maximum dose of beta-blocker and after 3 years. They included: gender, NYHA/Ross functional class, pharmacological treatment, blood pressure (BP), HR and echocardiographic parameters including LVEF, left ventricular systolic (LVESd) and diastolic diameter (LVEDd), time of follow up and outcomes including death and heart transplant (HT).

To assess the functional capacity in children, we used the following reported Ross Classification: class I included patients with no limitations or symptoms; II: infants with mild tachypnea or diaphoresis with feeding or children with mild to moderate dyspnea on exertion; III: infants presenting growth failure and marked tachypnea or diaphoresis with feeding or children with marked dyspnea on exertion; IV infants or children with symptoms at rest (Ross R.D., 2012). In patients older than 8 years we used NYHA class.

All statistical analysis was performed by SPSS Statistics 21 (IBM Corporation, Armonk, NY, United States). Categorical variables are expressed as absolute numbers or percentages. Continuous variables are presented as mean value and standard deviation (SD). Chi square analysis and t-student test were used to compare categorical and continuous variable, as appropriate. Regression analysis was performed to correlate changes in HR with changes in echo parameters, results were graphically represented. *p*-value was considered significant when ≤0.05.

## Results

**FIGURE 2:**
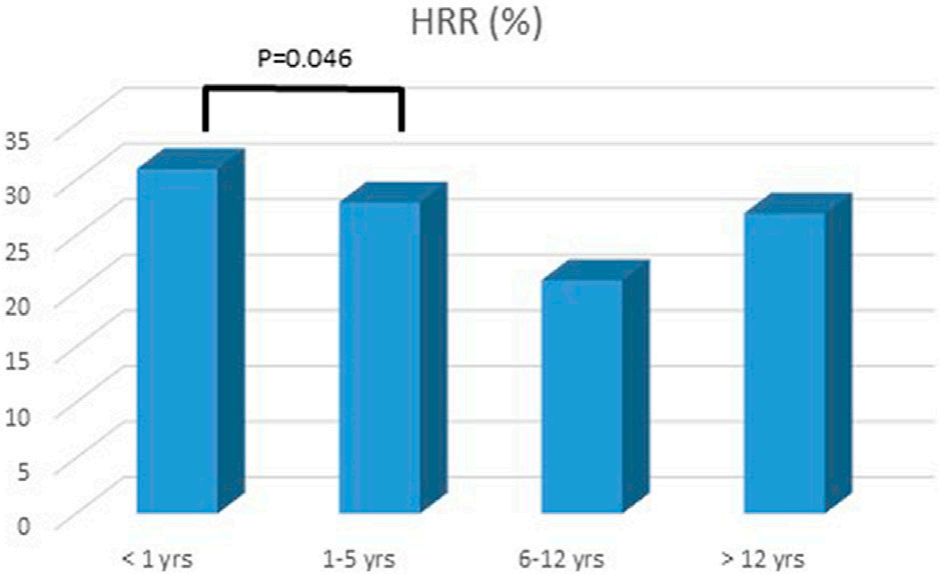
Heart rate reduction and age response. The group <1 year showed a significant response to 0.5 mg/kg/dose BID of carvedilol.

**FIGURE 3:**
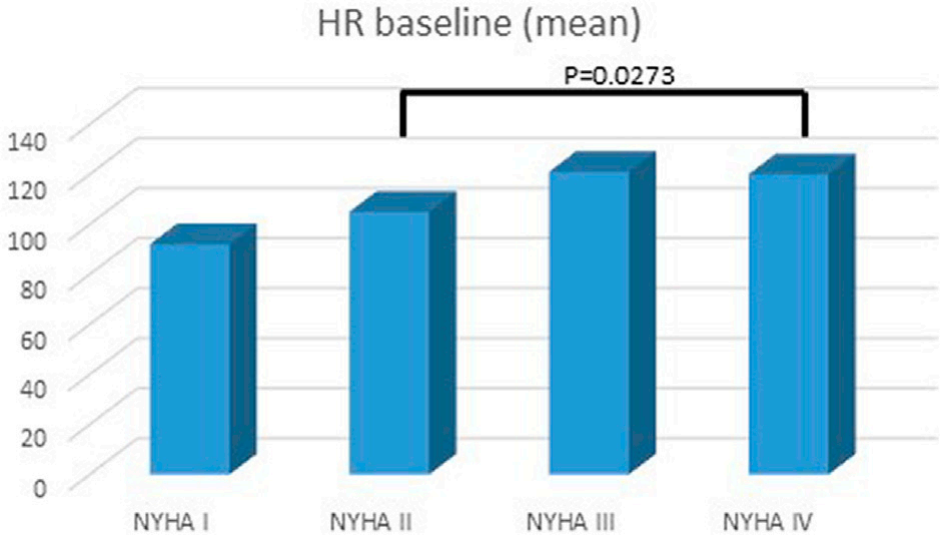
Heart rate and NYHA/Ross class at baseline. Highest heart rate was in the IV NYHA/Ross group.

A total of 100 patients were enrolled in the study: male 51%, age at diagnosis was 7 ± 8 years, age at last follow up was 12 ± 8.6 years, mean follow up was 4 ± 2.6 years (Table 1). At presentation, most patients were in NYHA/Ross class II or III. Mean BNP level was 422.6 ± 956 pg/ml. Mean HR at presentation was 108.8 ± 27.7 bpm. Basal HR was higher in patients in III and IV NYHA/Ross class and lower in I NYHA/Ross class (Figure 2). After reaching maximum tolerated dose mean HR was 76.9 ± 15.8 with an HR reduction (HRR) of 28 ± 11% compared to baseline (*p* < 0.05) (Table 2). No gender related differences in HRR have been identified as statistically significant (M: 29 ± 10%; F: 26 ± 12% *p*: 0.05). According to the age, we subdived all patients in four groups: < 1 year, between 1–5 years, 6–12 years, > 12 years <18 years. The analysis showed that there was a more pronounced effect of heart rate reduction in children below 1 year of age when compared to the group of 1–5 years (Figure 3).

**TABLE 1:** Clinical characteristics.

Total population (n)	100
**M (n)**	51
**Age (y) at diagnosis**	7 ± 8
**Age at last follow up (y)**	12 ± 8.6
**Follow up (y)**	4 ± 2.6
NYHA/Ross class at baseline (%)	
**I**	20
**II**	32
**III**	24
**IV**	17
Anti HF therapy (%)	
**Beta-blockers [carvedilol]**	100
**ACE inhibitors [captopril, enalapril, ramipril]**	100
**Anti Aldosteronics [spironolactone]**	42
**Diuretics [furosemide]**	85

**TABLE 2:** Effects of heart rate, Brain Natriuretic Peptide and echocardiographic parameters after up titration of carvedilol.

	Baseline	Maximum dose	*p*
**Heart Rate (bpm)**	108.8 ± 27.7	76.9 ± 15.8	*p* < 0.05
**BNP (pg/ml)**	422 ± 956	64 ± 129	*p* < 0.0001
**Echocardiographic Data**			
LVEDd (mm)	46 ± 16	38 ± 12	*p* = 0.2
LVESd (mm)	43.6 ± 16	35 ± 11	*p* = 0.08
LVEF	30.2 ± 10	45.9 ± 10.2	*p* < 0.0001

BNP, Brain Natriuretic Peptide; LVEDd, End Diastolic Diameter of Left Ventricle; LVESd, End Systolic Diameter of Left Ventricle; LVEF, Ejection Fraction of Left Ventricle.

**FIGURE 4:**
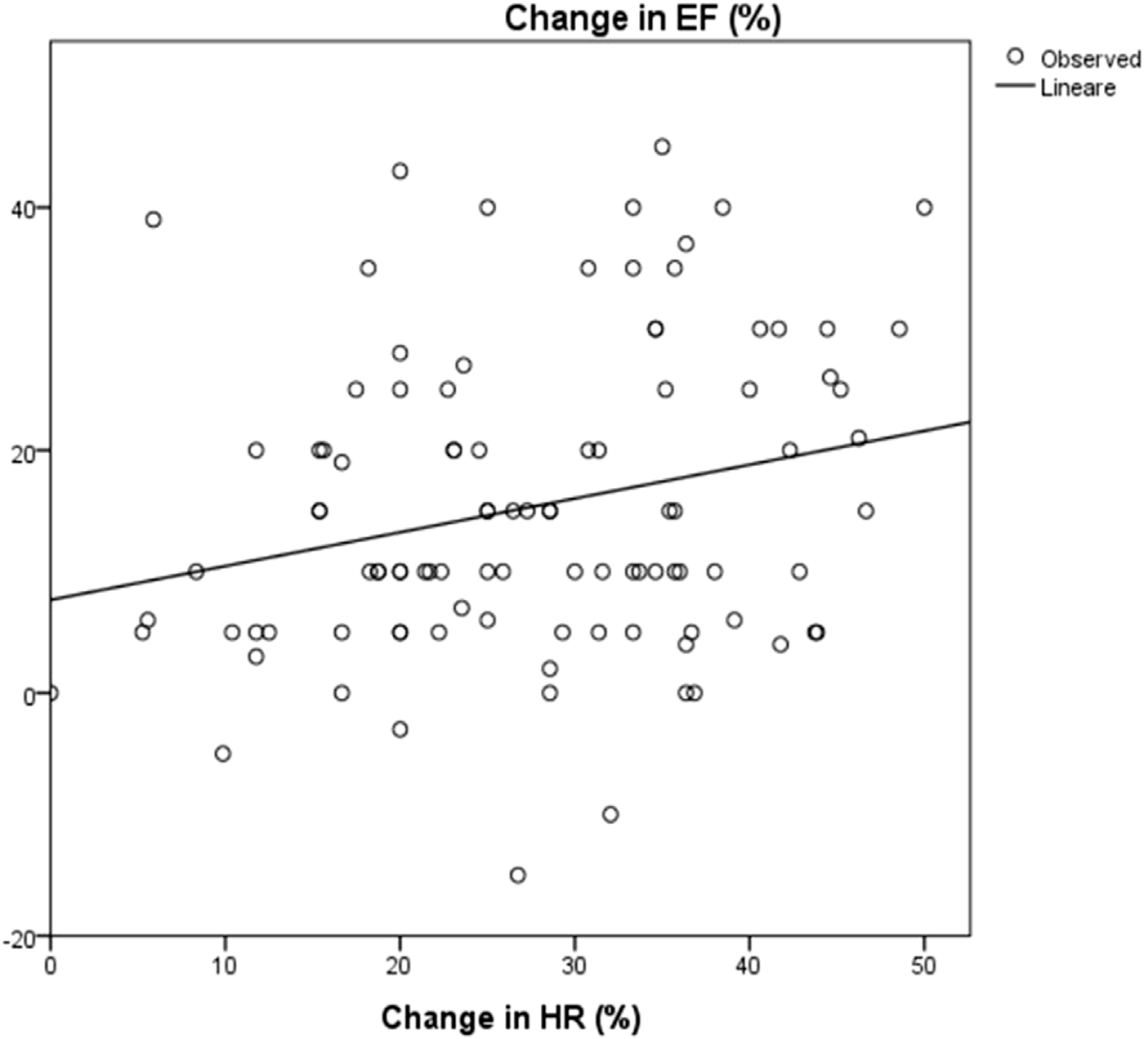
Correlation between change in HR and change in EF: R2 0.06; *p* = 0.014. HR: Heart rate; EF: Ejection Fraction. The change in ejection fraction was calculated between EF at baseline and after 3 years of follow up. The change in HR was calculated as the differences between baseline and at the maximum dose of carvedilol.

The mean LVEF before starting treatment was 30.2 ± 10%, at maximum therapeutic dose with HRR of more than 20%, LVEF increased by 13% with a mean EF of 43.7 ± 9.6% (*p* < 0.0001); after 3 years of follow up mean LVEF was 45.9 ± 10.2% (*p* < 0.0001), with an overall increase in EF of 15.7 ± 12.9% from baseline (Table 2). Regression analysis showed a statistically significant correlation between HRR and increase in LVEF (*R* ^2^ = 0.06, *p* = 0.014) (Figure 4).

**FIGURE 5:**
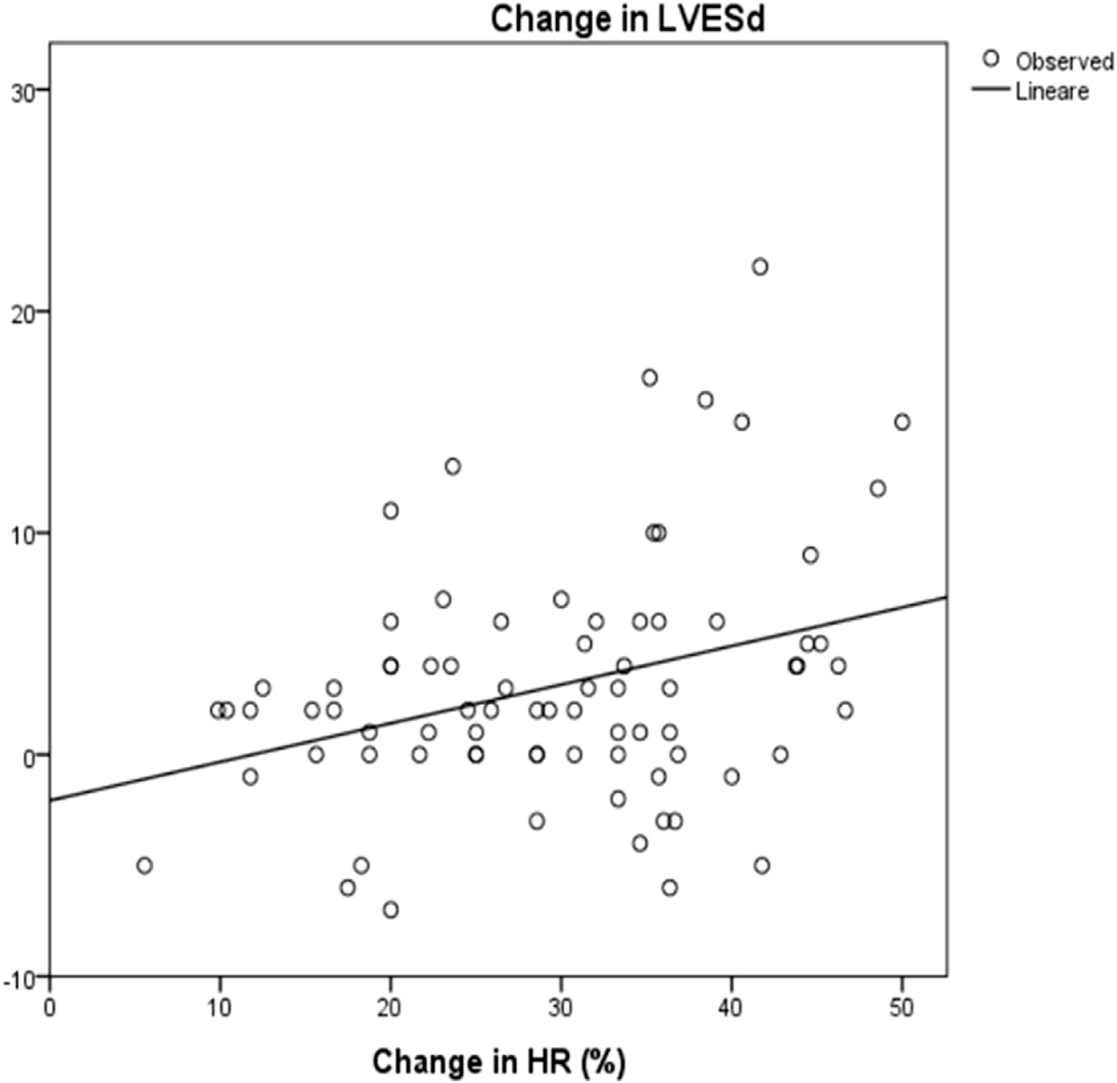
Correlation between change in HR and change in LVESd: R2 0.1; *p* = 0.003 HR: Heart rate; LVESd: Left Ventricular End Systolic Diameter. The change in end systolic diameter was calculated between baseline and after 3 years of follow up. The change in HR was calculated as the differences between baseline and at the maximum dose of carvedilol.

**FIGURE 6:**
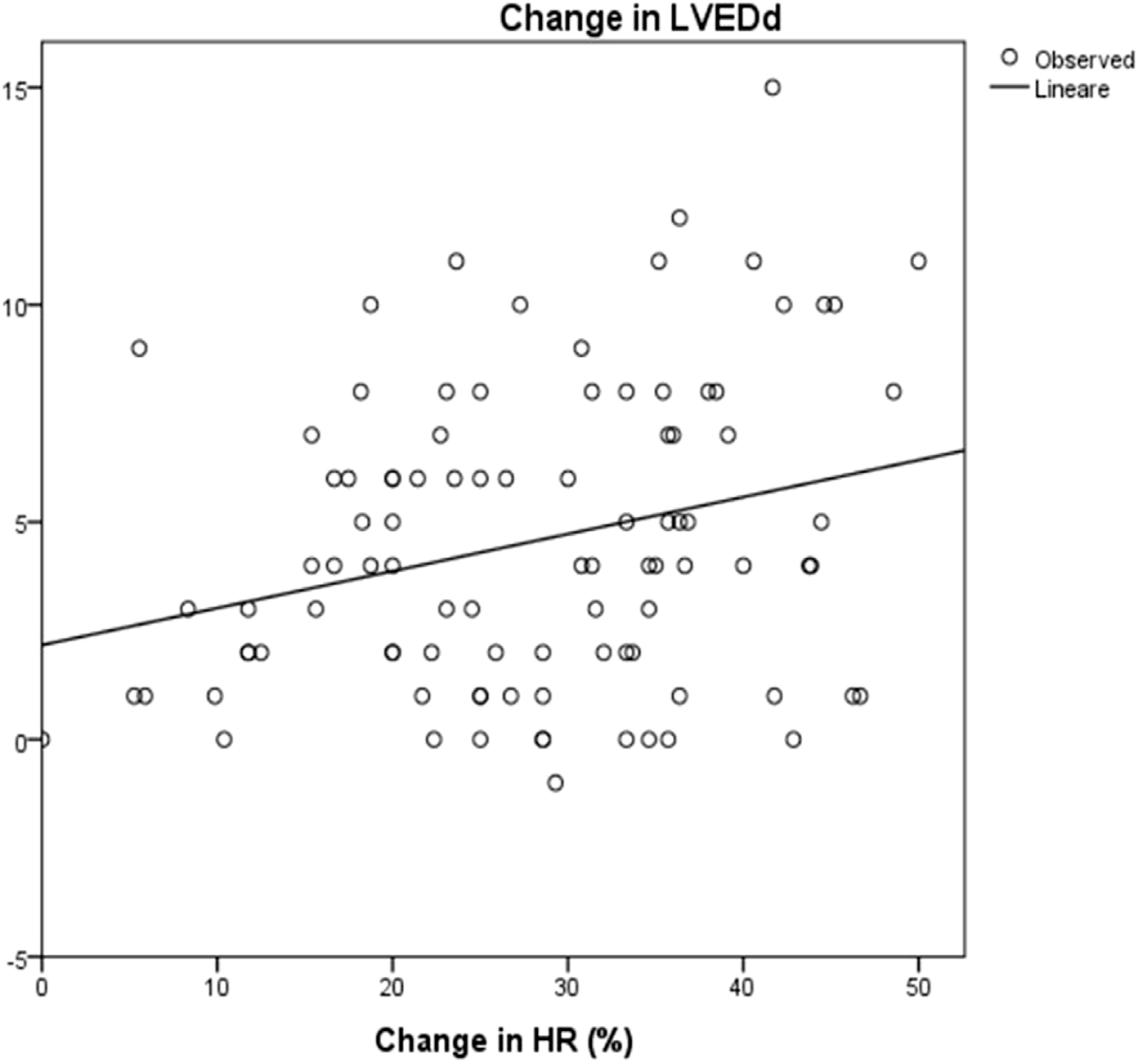
Correlation between change in HR and change in LVEDd: R2 0.07; *p* = 0.008 HR: Heart rate; LVESd: Left Ventricular End Diastolic Diameter. The change in end diastolic diameter was calculated between baseline and after 3 years of follow up. The change in HR was calculated as the differences between baseline and at the maximum dose of carvedilol.

Mean LVESd was 38 ± 12 mm at baseline and 35 ± 11 mm at the maximum dose (*p* = 0.08), with a reduction of around 4 ± 9 mm. Mean LVEDd at baseline was 46 ± 16 mm and 43,6 ± 16 mm at maximum carvedilol dose (*p* = 0,2), with a delta reduction of 2.7 ± 5.8 mm. LVEDs and LVEDd at baseline and at achievement of the maximum dose differed significantly in correlation to HRR (*R* ^2^ = 0.1, *p* = 0.003 and *R* ^2^ = 0.07, *p* = 0.008, respectively) (Figure 5 and Figure 6).

A HRR of >20% was positively related to an improvement in LVEF and to a reduction in LV diameters. No deaths or HT occurred during follow-up.

No episodes of hypotension and/or bradycardia were recorded during follow up.

No statistically significant relation in HRR have been found between gender at regression analysis (*p* = 0.05). Interestingly, HRR was more pronounced in patients aged <1 year, with a significant difference when compared to the older groups (*p* = 0.04).

## Discussion

This analysis demonstrates a clear relationship between reduction in HR with carvedilol and improvement of ventricular function in children affected by DCM and CHF. Regression analysis demonstrated that HR reduction is statistically related to a clinically important increase in LVEF. During long term FU, decreasing HR was also associated with a positive remodelling of LV. Importantly there were no adverse outcomes with this strategy.

To achieve these results, children required to reach a target dose of 1 mg/kg/day of carvedilol. This observation is also consistent with previous studies of pharmacokinetic and dynamics in children, showing that infants require higher dosages of carvedilol to achieve the same therapeutic levels of adults (Läer et al., 2002). Although the relation between HRR and use of beta-blockers may appear to be intuitive, in the real world this is challenging. HR is a mechanism of compensation in the acute setting. In this study all patients presented low level of BNP, confirming chronic HF. Therefore, our results should be interpreted only in this setting.

In adult, a metanalysis of thirty five trials showed that there is close relation between all-cause annualized mortality rate and HR (Flannery et al., 2008). A strong correlation between change in HR and change in LVEF was also observed. Current guidelines (McDonagh et al., 2021) stated that target HR should be 65 bpm. In pediatric CHF, the heterogeneous etiology of, age and individual response make it particularly challenging to assess whether the use of beta blockade is effective and safe. Recently, an analysis of Pediatric Cardiomyopathy Registry showed that elevated HR (2 or more SDs above the mean value adjusted for age) is independently associated with death (adjusted HR 2.6; *p* < 0.001) and with death or transplant (adjusted HR 1.5; *p* = 0.01) in children affected by DCM. An elevated HR was also associated to congestive HF, the need of anticongestive therapy, lower LVEF and greater LV dimension (Rossano et al., 2020).

However no data are currently available on how much it is necessary to lower HR to obtain a significant improvement in terms of prognosis and/or ventricular function. Unfortunately, HR was not considered as a therapeutic target in studies of betablockers in pediatric population. The Pediatric Carvedilol Trial failed to support beneficial effect of carvedilol on composite end-point of death, worsening symptoms of HF and rehospitalization (Shaddy et al., 2007). It should be noted that plasma concentrations of carvedilol were lower than the efficacy threshold derived in adults, HR decreased from baseline by about 10%. In this trial, the carvedilol group age ranged between 13 months and 154 months. Moreover, the population was heterogeneous for diagnosis (DCM and congenital heart disease). Notwithstanding, patients treated with higher dosage showed a trend to significance for DCM. Our data also showed that also in children <1 year of age, the effect of heart rate reduction is able to induce a long term effect and induce a positive LV remodelling. Usually, lower the heart rate in infant arise many concerns because of possible detrimental effect on cardiac output. Our data showed that also a reduction of 30% from baseline is beneficial in infants <1 year. These data are also sustained by PK/PD study by (Läer et al., 2002): the highest dosage (3 mg/kg/d) was required in infants to achieve the same therapeutic level of adult population.

Recently the availability of ivabradine in pediatric formulation raised the question of the impact of the HR in pediatric population. In a randomized, double-blind, placebo-controlled, phase II/III trial in children with symptomatic chronic HF due to DCM, using ivabradine, a HR reduction >20% was considered as a therapeutic target (Bonnet, et al., 2017). Ivabradine was prescribed in children older than 6 months. Ivabradine use was significantly associated with increased LVEF, at 6 months and 1-year follow-up. NYHA/Ross functional class improvement also showed a favourable trend. To note, in this study there were different threshold of HR according to the age. Lower-limit values were pre-specified, because most patients were already receiving beta-blockers. The minimal resting heart rate at inclusion for 5–18 year-olds was fixed at ≥70 beats/min based on studies in adults. The use of beta blocker was allowed as a concomitant treatment but no data on mean dosage were reported. Noticeably, HR reduction was safe: the frequency of adverse events was similar in both treatment groups (*p* = 1) with cardiac concerns reported less frequently by patients on ivabradine than placebo (15% vs. 31%, respectively). Interestingly, a high inter-individual variability in response to ivabradine was observed. This study showed that the HR might be manipulated also in children (Bonnet et al., 2017).

In pediatric HF, this report, in addition to the Ivabradine trial, strongly suggests that HRR is important. The current dosing of beta-blockers is empirical and not guided by clinical markers other than when adverse effects occur. The HR is an easy clinical marker to guide therapy and the HRR correlates closely with improvement in ventricular function. It is important to notice, that it is crucial to differentiate acute from chronic phase. Because the detrimental effect on systemic vascular resistance, beta blockers are contraindicated in the acute phase. Signs and symptoms could lack of accuracy to differentiate between acute and chronic status. The use of natriuretic peptide helped to identify these settings and BNP levels have been used to rule out those in acute phase. Plasma concentrations of natriuretic peptides can help to understand the individual risk of the patient and address therapy.

## Conclusion

This analysis shows that it is possible to utilize a simple clinical marker in the management of chronic HF with therapy including carvedilol in an individualized manner to improve LV function. HRR correlates with improved ventricular function and so in children, HR should be considered a potentially modifiable clinical marker to maximise therapy.

## Data Availability

The raw data supporting the conclusions of this article will be made available by the authors, without undue reservation.

## References

[B1] Bonnet D. Berger F. Jokinen E. Kantor P. F. Daubeney P. E. F. (2017). Ivabradine in children with dilated cardiomyopathy and symptomatic chronic heart failure. J. Am. Coll. Cardiol. 70 (10), 1262–1272. 10.1016/j.jacc.2017.07.725 28859790

[B2] Flannery G. Gehrig-Mills R. Billah B. Krum H. (2008). Analysis of randomized controlled trials on the effect of magnitude of heart rate reduction on clinical outcomes in patients with systolic chronic heart failure receiving beta-blockers. Am. J. Cardiol. 101 (6), 865–869. 10.1016/j.amjcard.2007.11.023 18328855

[B3] Fox K. Borer J. S. Camm A. J. Danchin N. Ferrari R. Lopez Sendon J. L. (2007). Heart Rate Working GroupResting heart rate in cardiovascular disease. J. Am. Coll. Cardiol. 50 (9), 823–830. 10.1016/j.jacc.2007.04.079 17719466

[B4] Fox K. Ford I. Steg P. G. Tendera M. Robertson M. Ferrari R. (2008). Heart rate as a prognostic risk factor in patients with coronary artery disease and left-ventricular systolic dysfunction (BEAUTIFUL): A subgroup analysis of a randomised controlled trial. Lancet 372 (9641), 817–821. BEAUTIFUL Investigators. 10.1016/S0140-6736(08)61171-X 18757091

[B5] Läer S. Mir T. S. Behn F. Eiselt M. Scholz H. Venzke A. (2002). Carvedilol therapy in pediatric patients with congestive heart failure: A study investigating clinical and pharmacokinetic parameters. Am. Heart J. 143 (5), 916–922. 10.1067/mhj.2002.121265 12040358

[B16] Lechat P. (1998). Beta-blocker treatment in heart failure. Role of heart rate reduction. Basic Res Cardiol Suppl. 93 (1), 148–155. 10.1007/s003950050243 9833143

[B6] McAlister F. A. Wiebe N. Ezekowitz J. A. Leung A. A. Armstrong P. W. (2009). Meta-analysis: Beta-blocker dose, heart rate reduction, and death in patients with heart failure. Ann. Intern. Med. 150 (11), 784–794. 10.7326/0003-4819-150-11-200906020-00006 19487713

[B7] McDonagh T. A. Metra M. Adamo M. Gardner R. S. Baumbach A. Bo¨hm M. (2021). 2021 ESC Guidelines for the diagnosis and treatment of acute and chronic heart failure. Eur. Heart J. 42, 3599–3726. 10.1093/eurheartj/ehab368 34447992

[B8] Packer M. Coats A. J. Fowler M. B. Katus H. A. Krum H. Mohacsi P. (2001). Carvedilol prospective randomized cumulative survival study GroupEffect of carvedilol on survival in severe chronic heart failure. N. Engl. J. Med. 344 (22), 1651–1658. 10.1056/NEJM200105313442201 11386263

[B9] Packer M. Fowler M. B. Roecker E. B. Coats A. J. Katus H. A. Krum H. (2002). Carvedilol prospective randomized cumulative survival (COPERNICUS) study GroupEffect of carvedilol on the morbidity of patients with severe chronic heart failure: Results of the carvedilol prospective randomized cumulative survival (COPERNICUS) study. Circulation 106 (17), 2194–2199. 10.1161/01.cir.0000035653.72855.bf 12390947

[B10] Packer M. Chalon S. Cucherat M. Arab T. Boissel J. P. (1998). Clinical effects of beta-adrenergic blockade in chronic heart failure: A meta-analysis of double-blind placebo-controlled randomized trials. Circulation 98 (12), 1184–1191. 10.1161/01.cir.98.12.1184 9743509

[B11] Pinto Y. M. Elliott P. M. Arbustini E. Adler Y. Anastasakis A. Böhm M. (2016). Proposal for a revised definition of dilated cardiomyopathy, hypokinetic non-dilated cardiomyopathy, and its implications for clinical practice: A position statement of the ESC working group on myocardial and pericardial diseases. Eur. Heart J. 37 (23), 1850–1858. 10.1093/eurheartj/ehv727 26792875

[B12] Ross R. D. (2012). The Ross classification for heart failure in children after 25 years: A review and an age-stratified revision. Pediatr. Cardiol. 33 (8), 1295–1300. 10.1007/s00246-012-0306-8 22476605

[B13] Rossano J. W. Kantor P. F. Shaddy R. E. Shi L. Wilkinson J. D. Jefferies J. L. (2020). Elevated heart rate and survival in children with dilated cardiomyopathy : A multicenter study from the pediatric cardiomyopathy Registry. J. Am. Heart Assoc. 9 (15), e015916. 10.1161/JAHA.119.015916 32750307 PMC7792277

[B14] Shaddy R. E. Boucek M. M. Hsu D. T. Boucek R. J. Canter C. E. Mahony L. (2007). Pediatric carvedilol study GroupCarvedilol for children and adolescents with heart failure: A randomized controlled trial. JAMA 298 (10), 1171–1179. 10.1001/jama.298.10.1171 17848651

[B15] Swedberg K. Komajda M. Bohm M. Borer J. S. Ford I. Dubost-Brama A. (2010). SHIFT InvestigatorsIvabradine and outcomes in chronic heart failure (SHIFT): A randomised placebo-controlled study. Lancet 376 (9744), 875–885. 10.1016/S0140-6736(10)61198-1 20801495

